# Prediction and Validation of Hub Genes Associated with Colorectal Cancer by Integrating PPI Network and Gene Expression Data

**DOI:** 10.1155/2017/2421459

**Published:** 2017-10-25

**Authors:** Yongfu Xiong, Wenxian You, Rong Wang, Linglong Peng, Zhongxue Fu

**Affiliations:** Department of Gastrointestinal Surgery, The First Affiliated Hospital of Chongqing Medical University, Chongqing 400016, China

## Abstract

Although hundreds of colorectal cancer- (CRC-) related genes have been screened, the significant hub genes still need to be further identified. The aim of this study was to identify the hub genes based on protein-protein interaction network and uncover their clinical value. Firstly, 645 CRC patients' data from the Tumor Cancer Genome Atlas were downloaded and analyzed to screen the differential expression genes (DEGs). And then, the Kyoto Encyclopedia of Genes and Genomes pathway enrichment analysis was performed, and PPI network of the DEGs was constructed by Cytoscape software. Finally, four hub genes (CXCL3, ELF5, TIMP1, and PHLPP2) were obtained from four subnets and further validated in our clinical setting and TCGA dataset. The results showed that mRNA expression of CXCL3, ELF5, and TIMP1 was increased in CRC tissues, whereas PHLPP2 mRNA expression was decreased. More importantly, high expression of CXCL3, ELF5, and TIMP1 was significantly associated with lymphatic invasion, distance metastasis, and advanced tumor stage. In addition, a shorter overall survival was observed in patients with increased CXCL3, TIMP1, and ELF5 expression and decreased PHLPP2 expression. In conclusion, the four hub genes screened by our strategy could serve as novel biomarkers for prognosis prediction of CRC patients.

## 1. Introduction

Colorectal cancer (CRC) is one of the most common malignancies and an important contributor to cancer mortality and morbidity [1, 2]. Based on the previous studies, CRC is well known as a heterogeneous disease in which aberrant expression of hub genes mediated tumor initiation, progression, and metastasis [3]. A series of researches have demonstrated that hub genes in CRC, such as DHRS9 [4], GRIM-19 [5], EphA2 [6], and STYK1 [7], not only are involved in regulating a variety of cellular processes including cell proliferation, survival, differentiation, migration, and apoptosis but are also correlated with disease progression and prognosis of patients with CRC. However, due to the labor-intensive and inefficiency method, only small part of CRC-related hub genes has been investigated using traditional detection methods for individual biomarker. Besides that, the results from these methods were not uniform and shared only a limited degree of overlap. All these facts indicate that it is necessary to seek a new approach to screen hub genes effectively and accurately.

Protein-protein interactions (PPIs) are crucial for biological processes including gene expression, cell growth, proliferation, and apoptosis [8, 9]. Numerous studies have implicated that aberrant PPIs were the basis of multiple aggregation-related diseases, especially involved in cancer occurrence and progression [10–13]. Moreover, it has been clarified that proteins expression is a dynamic process as their functions tend to be regulated in network [14]. Understanding protein interactions provides an efficient approach for screening hub genes. Thus, due to the potential significance of PPI network in cancer biology, its implication in human malignancies has aroused increasing attention. Hub genes identified by PPI network based approach have been reported in cancers of breast [15] and liver [16] and gastric cancer [13]. Furthermore, the PPI network has a fraction of highly connected region (subnet) with high probabilities of engaging in essential biological regulation [17], whereas those lightly connected nodes do not exert critical role in the whole network's integrity [17, 18]. Besides that, gene silencing experiment further confirmed that intramodular hubs were significantly associated with disease status [19]. Thus, hub genes obtained from PPI subnet were more meaningful than individual genes screened without network information [20]. More importantly, the identified hub genes achieved higher accuracy than individual genes in the classification of patients with different clinicopathologic features such as metastatic versus nonmetastatic patients [21, 22].

However, there are common limitations in previous studies. Firstly, due to many factors including small sample size, different platforms, and unpaired methods for data processing, the differential expression genes (DEGs) as the essential data for PPI network analysis are not consistent in different studies.  Moreover, former studies almost did not validate the identified hub genes by experiment and did not reveal their clinicopathologic correlation and prognostic value. Therefore, we aimed to identify the hub genes based on PPI network approach in a large number of CRC cohorts downloaded from TCGA and then further validate and investigate their clinical values.

## 2. Results

### 2.1. Integrated RNA-Seq Data and Clinical Information in the TCGA CRC Cohort

**Figure 1:**
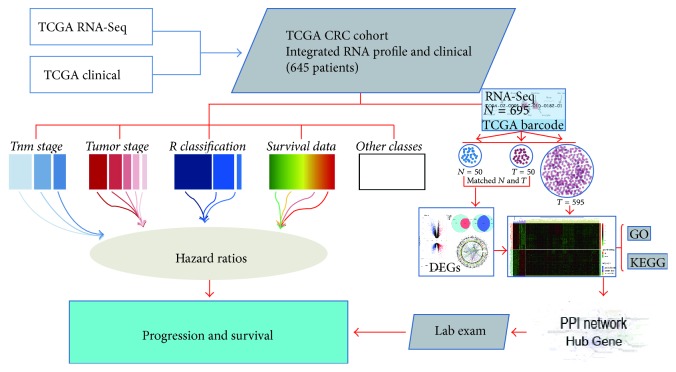
*Flowcharts for deriving and validating hub genes in CRC*. Expression profiles and corresponding clinical data for 645 CRC patients were obtained from The Cancer Genome Atlas (TCGA) data portal (July 2016). PPI network analysis based on differential expression genes between paired sets (normal and adjacent tumor tissue) was used to identify hub genes. Subsequently, patients with complete clinical information were included in the study to investigate the clinicopathology features and prognostic value of the identified hub genes.

**Figure 2:**
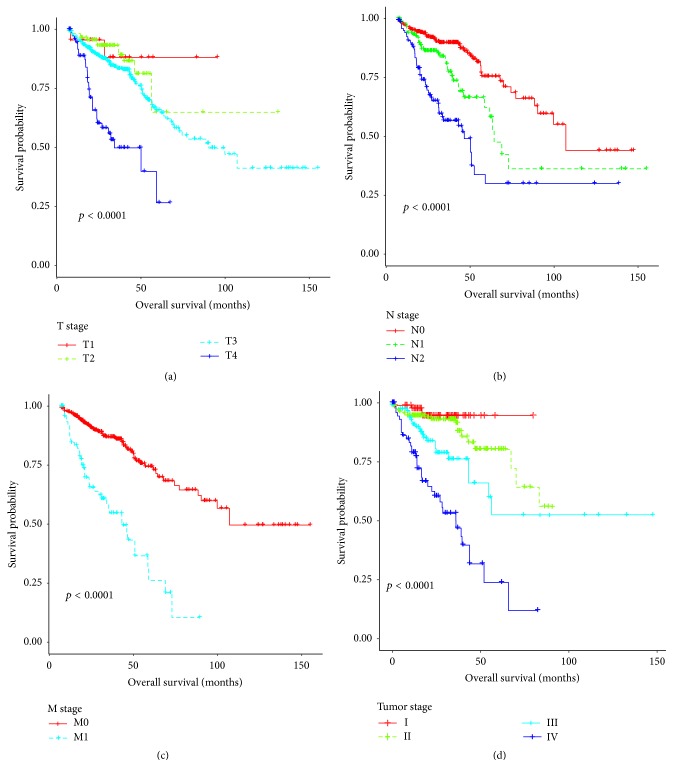
*Kaplan-Meier survival estimates by the TNM (Tumor, Node, Metastasis) system and tumor stage in CRC cohort*. (a–c) TNM stage (overall log-rank test, *p* < 0.0001). (d) Tumor stage (overall log-rank test, *p* < 0.0001).

We first downloaded all of the publicly available RNA-Seq data and clinical information from TCGA before July 30, 2016, through GDC Data Transfer Tool [22]. The tool provides a standard client-based mechanism in support of high performance data downloads and submissions. After preprocessing, a huge genes expression matrix formed by a total of 695 RNA-Seq sets of data from 645 CRC patients was obtained. TCGA barcode for RNA-Seq and clinical information in different data files was used to associate those data tables. Finally, a large scale of gene expression data in paired CRC tumor and corresponding adjacent normal tissues were obtained by analyzing the information extracted from barcode (see flowchart to Figure 1 for details). Before studying the clinical significant of hub genes, Kaplan-Meier test was performed to assess the relationship between clinicopathologic features and prognosis in the TCGA CRC cohort. The preliminary assessment revealed that local invasion (T stage), lymph node metastasis (N stage), distal metastasis (M stage), and TNM stage were significantly associated with prognosis, which is consistent with previous reports [23]. Thus, the clinical information was suitable for the next study (Figure 2).

### 2.2. Identification and Functional Enrichment Analysis of DEGs

**Figure 3:**
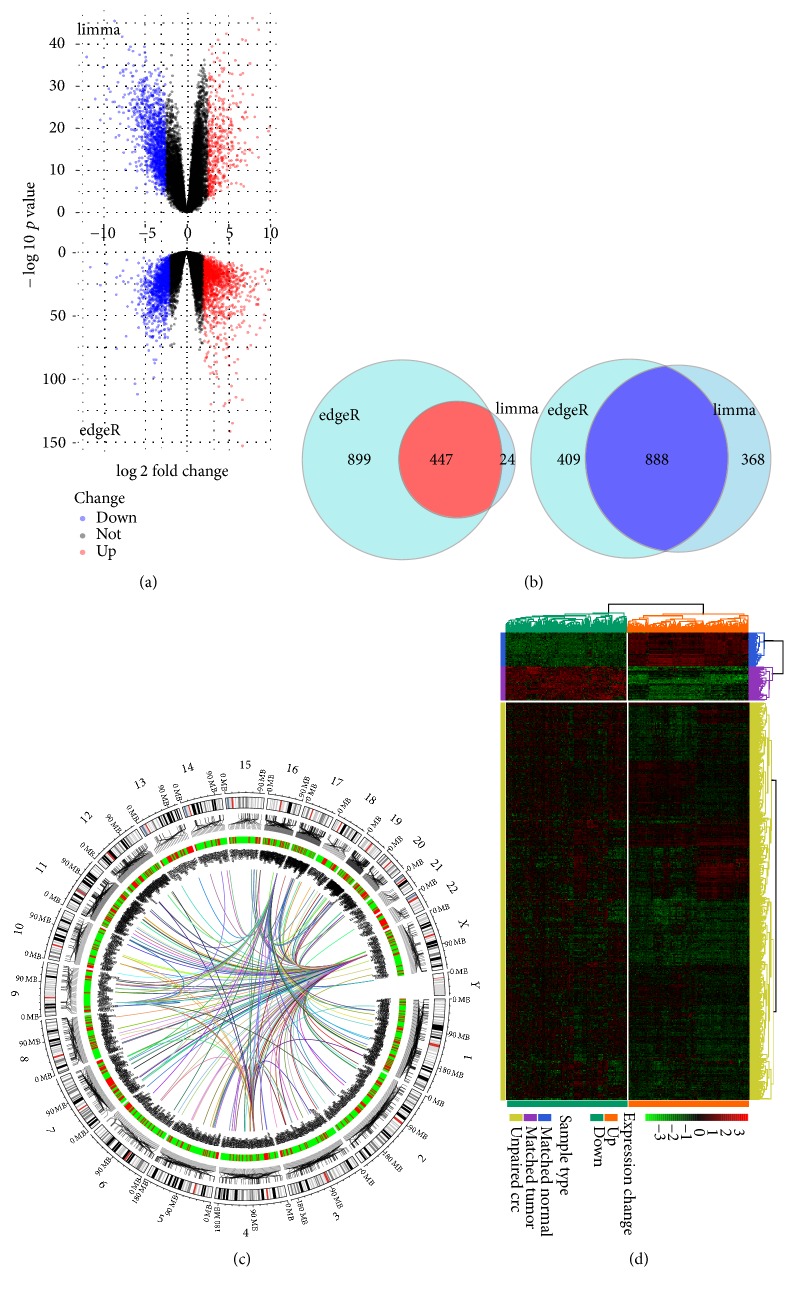
*Screening of gene signatures and identification of DEGs*. (a) Volcano Plot visualizing the DEGs of CRC was screened by limma and edgeR. The red and blue points in plot represent the differentially expressed genes with statistical significance (*p* < 0.05, |log⁡FC| ≥ 2). (b) Venn diagram demonstrated the intersection set of upregulated (red) and downregulated (blue) genes identified by limma and edgeR, respectively. (c) Circos plots showing the distribution and expression change of DEGs. (d) Heatmap of the significant differentially expressed genes. The right longitudinal axis showed the clustering information of samples. The samples were mainly divided into three major clusters and these three clusters were the adjacent normal tissue (*N* = 50), paired tumor tissue (*N* = 50), and the other CRC tumor tissue (*N* = 595); the left longitudinal axis showed the clustering information of DEGs. Red represents the upregulated genes, while green represents the downregulated genes.

To investigate the DEGs associated with CRC, expression files of 50 paired human primary colorectal cancer and corresponding adjacent normal tissues were integrated to screen DEGs using edgeR [24] and limma [25], respectively. The criteria of |log⁡FC| ≥ 2 and *p* ≤ 0.05 were used to determine the significant DEGs (Figure 3(a)). For eliminating the potential error, up- and downregulated gene sets were intersected to obtain optimal DEGs (Figure 3(b)). Eventually, a total of 1335 DEGs were screened, among which 447 genes were upregulated while 888 genes were downregulated in cancer tissues. In order to encapsulate the DEGs in an all-round way, Circos Plots were created to display the distribution, correlation, and variation of DEGs in chromosome (Figure 3(c)). In addition, the relative mRNA expression levels of DEGs among paired tumor and adjacent normal and unpaired tumor tissue were analyzed and compared (Figure 3(d)). By assigning different colors to individual DEGs, the expression pattern of DEGs was clearly displayed. Obviously, the consistency of DEGs expression levels within the paired tumor group was higher than unpaired tumor group (Figure 3(d)). These findings at least in part account for the accuracy of the DEGs analysis conducted in the present study.

**Figure 4:**
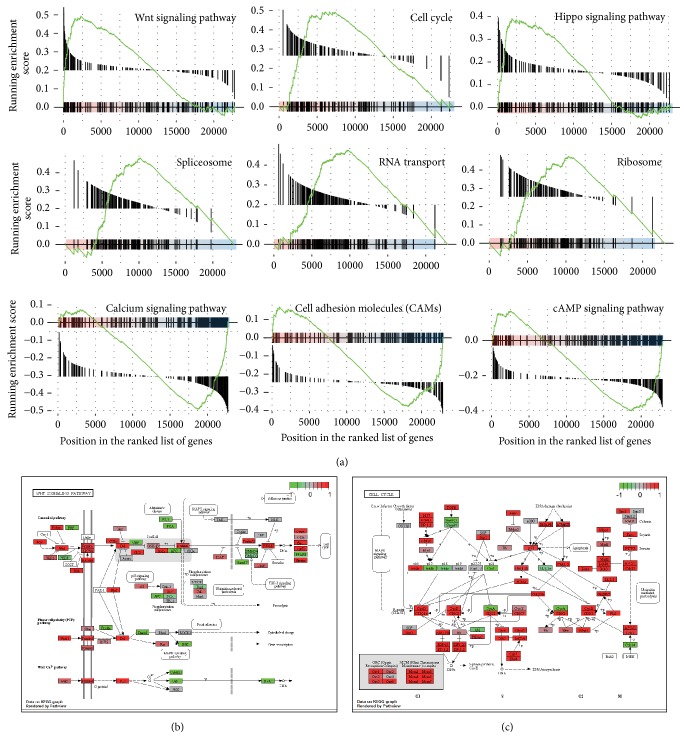
*Pathway enrichment analysis of DEGs*. (a) Enrichment plots for significant pathways identified by GSEA include Wnt signaling pathway, cell cycle, Hippo signaling pathway, spliceosome, RNA transport, ribosome, calcium signaling pathway, cell adhesion molecules (CAMs), cGMP-PKG signaling pathway, and cAMP signaling pathway. (b-c) The signaling pathway of Wnt and cell cycle (this image was obtained by Kyoto Encyclopedia of Genes and Genomes with permission). Red represents the upregulated genes, while green represents the downregulated genes.

Furthermore, KEGG enrichment analysis was conducted to interpret biological meanings of DEGs (Figure 4(a)). 9 pathways were significantly enriched including Wnt signaling pathway (*p* = 1.65 × 10^−3^), cell cycle (*p* = 1.67 × 10^−3^), Hippo signaling pathway (*p* = 9.88 × 10^−3^), spliceosome (*p* = 1.66 × 10^−3^), RNA transport (*p* = 1.63 × 10^−3^), ribosome (*p* = 1.66 × 10^−3^), calcium signaling pathway (*p* = 2.56 × 10^−3^), cell adhesion molecules (CAMs) (*p* = 5.01 × 10^−3^), cGMP-PKG signaling pathway (*p* = 2.55 × 10^−3^), and cAMP signaling pathway (*p* = 2.67 × 10^−3^) (Figure 4(a)). Besides that, almost all of the target genes of Wnt (Figure 4(b)) and cell cycle (Figure 4(c)) signaling pathway were upregulated, which play a critical role in the occurrence and development of carcinoma. Taken together, these results suggest that the DEGs screened in the present study are credible and consistent with previous knowledge of CRC.

### 2.3. Identification of Hub Genes by Using PPI Network

**Figure 5:**
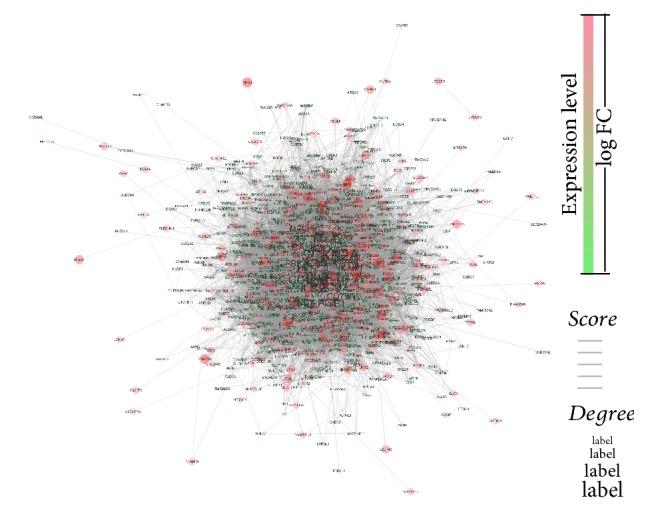
*PPI network of all DEGs*. Interactome of the 1335 genes showing 1166 nodes and 21671 edges in the PPI network encompassing four subnets in CRC. Genes were denoted as nodes in the graph and interactions between them were presented as edges. Green color indicates downregulated genes, and red color indicates upregulated genes; the label size represents the degree value; the thickness of connection line represents the level of closeness between two node.

**Figure 6:**
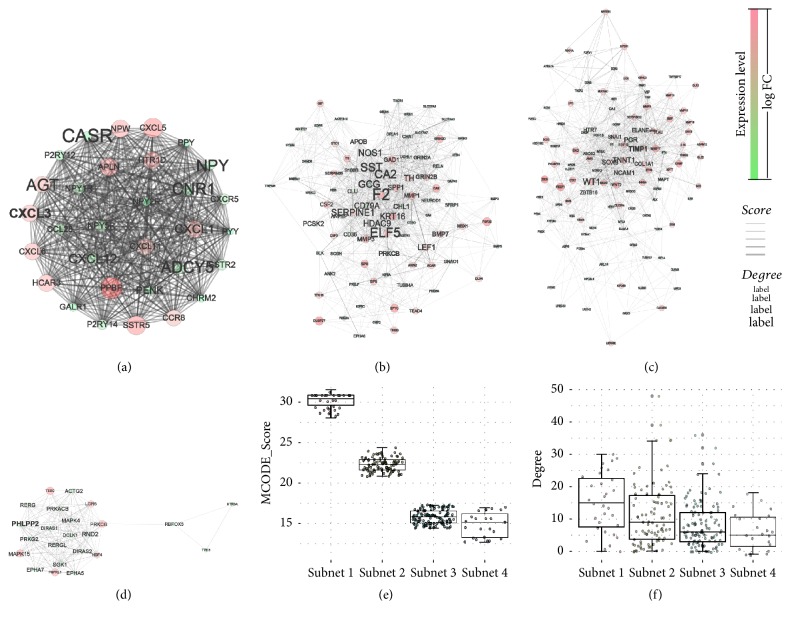
*Highly interconnected region form four best subnets among the 1335 DEGs and integrated centralities based analysis of subnet*. Green color indicates downregulated genes, while red color indicates upregulated genes; the label size represents the level of degree. (a–d) subnet 1, subnet 2, subnet 3, and subnet 4, respectively. (e-f) Comparisons of MCODE_score and degree centrality among the four subnets. There were significant differences in MCODE_score and degree distribution among each subnet.

To investigate the hub genes associated with CRC, the protein interaction data were downloaded from STRING database and a giant network was constructed by linking causal DEGs. The detail parameters were as follows: number of nodes = 1201, number of edges = 4090, and average node degree = 6.81. The size of label represents the degree index. The thickness of connection line indicates the level of closeness between two proteins. Color gradients from red to blue represent the change of log⁡FC (Figure 5). To find biologically essential subnets and corresponding hub genes, MCODE [26] plugin was used to investigate the whole network. Finally, four subnets in the original network were obtained according to the screening criteria at node score cut-off = 0.3, *K*-core = 4 (Figures 6(a)–6(d)). It is well demonstrated that disease related subnet (highly connected region) was often of clinical importance, and the hub genes among the subnets were involved in crucial biological processes [17]. The importance of each gene was evaluated by the centrality analysis from the four subnets including MCODE_score and degree. Obviously, there were some genes with significant differences in MCODE_score (Figure 6(e)) and degree (Figure 6(f)) among each subnet. These results demonstrated that the genes were highly interconnected within subnet while the subnets were independent of each other. Then those genes in each subnet with the highest MCODE_score were considered as the hub genes. In the present work, four hub genes were selected including CXCL3, ELF5, PHLPP2, and TIMP1.

### 2.4. Validation of CRC-Related Hub Genes and Its Association with Clinicopathologic Features

**Figure 7:**
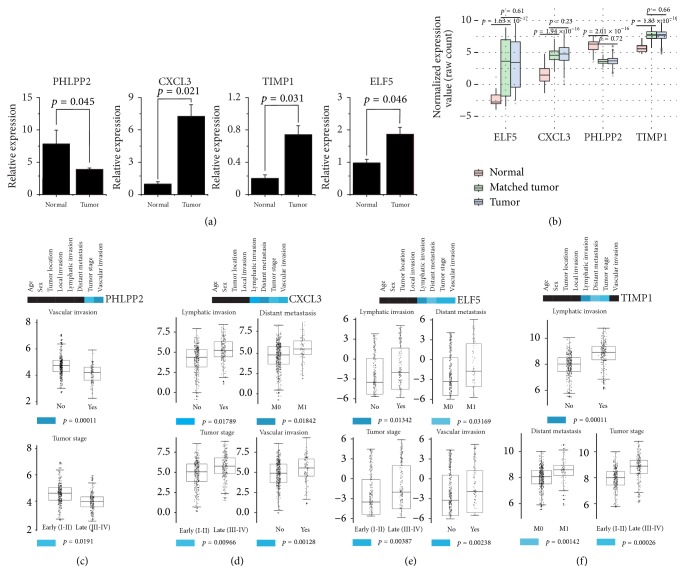
*Experiment validation and clinicopathologic features of CRC-related hub genes*. (a) qRT-PCR analysis of hub genes expression in the CRC tissues and the adjacent noncancerous tissue. (b) CRC-related hub genes expression in TCGA dataset. For boxplots, expression values of hub genes were normalized and box width was proportional to the square root of sample size in each variant. (c–f) Relationship between hub genes expression and clinicopathologic features. Color bar revealed the statistical significance of relationship between clinical features and hub gens: black represents *p* ≥ 0.05 and blue gradient represents *p* < 0.05 in which the deeper the color, the stronger the significant difference.

To determine the aberrant expression of CRC-related hub genes, qRT-PCR analysis was conducted in 25 pairs of cancerous and adjacent normal tissue derived from the same patient. As obviously shown in Figure 7(a), the mRNA expression of CXCL3, ELF5, and TIMP1 significantly increased in CRC tissues compared with matched adjacent normal tissues, whereas PHLPP2 mRNA expression was decreased in CRC tissues; and these results were further confirmed by TCGA database (Figure 7(b)).

To investigate the relationship between the expression levels of hub genes and clinicopathologic features, 498 CRC patients with complete clinical information were summarized in Table 1 and were selected to conduct correlation analysis. As shown in Figures 7(c)–7(f), high expression of CXCL3, ELF5, and TIMP1 was significantly associated with lymphatic invasion, distance metastasis, and advanced tumor stage. Besides that, high CXCL3 and ELF5 expression were also significantly related to vascular invasion. Moreover, low PHLPP2 expression was significantly related to vascular invasion and tumor stage. However, there were no significant associations observed between the four hub genes expression and clinical characteristics such as age, sex, and tumor location.

### 2.5. Prognostic Values of Hub Genes for Patients with CRC

**Figure 8:**
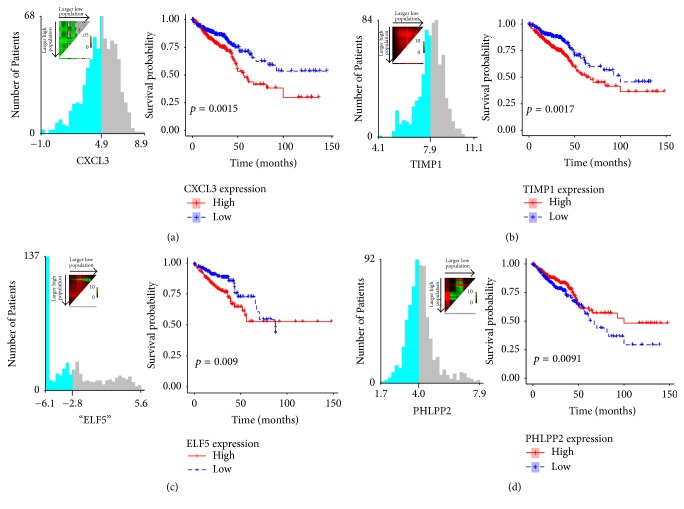
*Clinical significance of hub genes*. (a–d) Kaplan-Meier survival analysis by X-tile plots cut-off point. (a) CXCL3, *p* < 0.0015. (b) TIMP1, *p* < 0.0017. (c) ELF5, *p* < 0.43. (d) PHLPP2, *p* < 0.0096. X-tile plots are shown in the left panels. The plot showed the chi-squared log-rank values created when the cohort was divided into two groups. The optimal cut-point highlighted by the color variations in the left panels is shown on a histogram of the entire cohort and a Kaplan-Meier plot (right panels).

To further investigate the clinical outcomes of hub genes for patients with CRC, X-tile [27] and Kaplan-Meier survival analysis were performed to evaluate the effects of hub genes on overall survival (OS) (Figure 8). The results showed that CRC patients with high CXCL3 (*p* = 0.0015), ELF5 (*p* = 0.0094), and TIMP1 (*p* = 0.0017) expression had a significantly shorter OS (Figures 8(a)–8(d)), whereas, for patients with low PHLPP2 expression, a longer OS was observed (*p* = 0.0091).

## 3. Discussion

CRC is a disease caused by cumulative genetic, epigenetic, somatic, and endocrine aberrations [28]. Understanding the molecular mechanism of CRC is of critical importance for CRC diagnosis and treatment. Since microarray and high-throughput sequencing provide expression levels of thousands of genes in human genome simultaneously, it has been widely used to predict the potential therapeutic targets for CRC [29–31]. However, gene expression profiling about DEGs showed markedly different in previous studies. For example, Liang et al. [11] identified 3500 DEGs in CRC including 1370 upregulated genes and 2130 downregulated genes. In contrast, another research which also focused on DEGs showed a total of 4937 differentially expressed genes, among which 2974 genes were upregulated while 1963 genes were downregulated in cancer samples [32]. These inconsistencies about DEGs in different studies at least in part may be caused by the small sample size and methodological differences in the preprocessing. At present, several studies have demonstrated that the low replication of biological samples did not correctly screen DGEs because of its insufficient statistical power [33] and also did not preciously investigate the natural biological variability [34]. Recent researches further indicated that at least 12 replicates were essential to identify the major DEGs in RNA-seq experiment. Furthermore, in order to make the result cover more than 85% of all DEGs, 20 biological replicates were a prerequisite [35]. Obviously, many previous studies did not yet meet the minimum standards. Although systematic review or integrated analysis was used to determine DEGs in cancer, such approach seems not rigorous due to the data from different platforms and the different methods for data processing. All these facts demonstrated that, in order to accurately identify DEGs based on a large scale of paired sample, it is essential to sequence and preprocess these data according to the same and high criteria discussed above.

Currently, it is clearly understood that screening a CRC-related DEG do not necessarily equate considering it as biologically meaningful. Thus, many effective methods such as GO and KEGG have been adopted to interpret the significance of DEGs, hoping to elucidate the role of individual molecule in various biological processes. However, there is still a problem that a large number of human genes have not yet been assigned to a definitive pathway based on enrichment analysis. The significance of these genes will thus not be detected in the identification of individual marker genes. Therefore, it is necessary to adopt more effective and accurate approaches in hub genes selection. Accumulating mechanism studies indicated that the essence of biological process is a strict and quantifiable interaction between countless biomolecules [36]. Various types of networks emerge from the sum of these interactions including PPI network [37], phosphorylation networks [38], and signaling and transcription regulatory networks [39]. Bionetwork studies, particularly the study of the PPI, provide an insight into the structures and the dynamics of the complex intercellular interactions. Based on the network theory, there were numbers of highly connected regions (subnet) with specific function, which is significant in the biological systems [40, 41]. More importantly, gene silencing experiments further confirmed that genes within highly connected regions tend to be significantly associated with disease status [20]. Hence, identification of hub genes based on network analysis was more meaningful than individual genes screened without network information [21].

Considering all the above reasons, we performed the present study. Firstly, we extracted the expression data from TCGA CRC cohort and identified 1335 DEGs between paired CRC and normal tissues, among which 447 genes were upregulated and 888 were downregulated in cancer tissues. Enrichment analysis showed that these DEGs were mainly involved in Wnt signaling pathway, cell cycle, RNA transport, and cell adhesion molecules. Of note, as the small sample size and methodological differences in the preprocessing lead to the major inconsistence between CRC-related expression signatures; in this study, the DEGs were obtained from a large number of paired TCGA CRC cohort and these data were preprocessed according to a strict and same criteria. Moreover, edgeR and limma were used to obtain candidate DEGs, respectively, and get intersection set. These methods ensured the reliable and accurate results.

Since a series of studies have reported that PPI network is of importance to identify the genes with potential clinical value, we therefore constructed CRC-related PPI network by the 1201 mapped DEGs from the STRING database. Subsequently, the top ranked genes within four highly connected regions were extracted as hub genes, which include CXCL3, ELF5, PHLPP2, and TIMP1. Our further experiment about validation and clinicopathologic analysis revealed that high expression of CXCL3, ELF5, and TIMP1 was significantly associated with lymphatic invasion, distance metastasis, and advanced tumor stage. Besides that, high CXCL3 and ELF5 expression was also significantly related to vascular invasion, and low PHLPP2 expression was significantly associated with vascular invasion and tumor stage. Similar to what we observed in the current study, a recent research demonstrated that CXCL3 was overexpressed in most cases of aggressive prostate and breast tumors, and its expression was associated with poor prognosis [42]. the upregulated ELF5 expression in endometrial carcinoma was also related to higher disease stage [43]. Moreover, low expression of PHLPP2 was significantly related to the advanced tumor stage, poor differentiation, and increased lymph node metastasis in patients with hypopharyngeal squamous cell carcinoma [44]. PHLPP2 was also described as a survival and proliferation related suppressor in various cancers [45]. More importantly, according to the findings of Song et al., the clinical feature of aberrant expression of TIMP1 in CRC patients was consistent with our results [46]. Taking together all these previous data combined with our present results, at least in part, confirmed that PPI network analysis is an effective method to identify hub genes with clinical significance.

Another important finding of the current study was the prognostic value of the hub genes in CRC. Survival analysis showed that CRC patients with high CXCL3, ELF5, and TIMP1 expression had a significantly shorter OS, whereas, for patients with low PHLPP2 expression, a longer OS was observed. These findings further validate the clinical significance of hub genes identified by PPI network. More significantly, besides the aberrant expression of TIMP1 being confirmed by previous study, it is a marker of clinical significance in the diagnosis and prognosis of patients with colon carcinomas [46]; to the best of our knowledge, the current study is the first to report the prognostic value of CXCL3, ELF5, and PHLPP2 in CRC.

In conclusion, our present study performed a PPI network analysis of differential expression signature between paired CRC and normal control to obtain hub genes. More importantly, our further clinicopathologic and prognostic investigation revealed that all of these hub genes were of important clinical significance. PPI network analysis was an effective method to identify hub genes with clinical significance. However, further clinical and mechanism studies focusing on these hub genes are required to uncover the underlying mechanisms in tumorigenesis of CRC.

## 4. Materials and Methods

### 4.1. Clinical Information and RNA-Seq Dataset in TCGA

RNA-Seq data and corresponding clinical data for 645 CRC patients were obtained from The Cancer Genome Atlas (TCGA) data portal (July 2016) [12]. Both the RNA-Seq data and clinical data including outcome and clinicopathologic information of TCGA CRC patients were deposited at the Data Coordinating Center (DCC), and these data are publicly available and open access. TCGA data are classified by data type and data level, to allow structured access to this resource with appropriate patient privacy protection. This study meets the publication guidelines provided by TCGA [13]. Samples and corresponding clinical data were cross-referenced by tumor barcodes. The patients were included in the study to meet the following criteria: (1) patients with fully clinical information (clinicopathological data and expression profiles); (2) patients documented overall survival.

### 4.2. Screening of Differential Expression Genes (DEGs) and Enrichment Analysis

To identify DEGs between CRC and normal tissues, the raw counts of expression data obtained from the TCGA dataset (645 CRC samples and 50 normal tissue) were normalized by a weighted trimmed mean of the log expression ratios. The batch effect was removed using a generalized linear model [39]. The expression differences were characterized by log⁡FC (log⁡2 fold change) and associated *p* values. The log⁡FC ≥ 2 and log⁡FC ≤ −2 with *p* < 0.05, respectively, represented upregulated and downregulated mRNAs. The analysis was performed using the R/Bioconductor package of limma (version 3.32.3) and edgeR (version 3.18.1). Eventually, the genes identified to be differential expression by both of the algorithms were selected as DEGs.

To further assess the signaling pathway of the gene signatures, we performed a pathway analysis based on Kyoto Encyclopedia of Genes and Genomes (KEGG) database (http://www.genome.jp/kegg/). DEGs were applied to this database in order to investigate the biological pathways that might be involved in the occurrence and development of CRC. The analyses were performed by clusterProfiler package (version 3.4.4). KEGG enrichment with parameter set as nPerm = 1000, minGSSize = 120, *p* < 0.05, was screened to further analysis.

### 4.3. PPI Network Construction and Analysis

The protein interaction data were selected from the Search Tool for the Retrieval of Interacting Genes/Proteins (STRING) 9.1 database and a network was constructed by linking causal disease genes with the selected gene signatures using Cytoscape 3.1.0, a free software package for visualizing, modeling, and analyzing the integration of biomolecular interaction networks with high-throughput expression data and other molecular states [47]. Subsequently, we investigated the substructure of the giant protein interaction network extracted from the above constructed network and focused on highly connected nodes known as subnet using the MCODE clustering algorithm [26], including vertex weighting, complex prediction, and optional postprocessing. The regulation relationships among various genes in each subnet were analyzed through calculating the topological properties of the network such as degree and MCODE_score. Moreover, highest interacting genes in each subnet were identified as hub genes.

### 4.4. Experimental Validation and Clinical Value of Hub Gene

To validate the results of integrated bioinformatics analysis, 25 pairs of fresh CRC and adjacent noncancerous tissues were obtained from 25 patients by experienced surgeons and examined by experienced pathologists at the First Affiliated Hospital of Chongqing Medical University between July and December, 2016. Written informed consent was obtained from all patients or their guardians. The samples were frozen immediately and stored until use. Then, quantification of hub genes was performed by real-time PCR as described previously [48]. For survival analysis, we used the Kaplan-Meier method to analyze the correlation between overall survival and the hub genes, and the log-rank test was used to compare survival curves. The optimum cut-off value for the hub genes using X-tile plots based on the association with mortality of the patients. X-tile plots provides a single and intuitive method to assess the association between variables and survival. The X-tile program can automatically select the optimum data cut-point according to the highest *χ*^2^ value (minimum *p* value) defined by Kaplan-Meier survival analysis and log-rank test [27]. We did the X-tile plots using the X-tile software version 3.6.1 (Yale University School of Medicine, New Haven, CT, USA).

## Figures and Tables

**Table 1:** Clinical features for the CRC patients in the TCGA cohort.

Characteristics	Number of patients (%)
	(*n* = 498)
Age (years)	
<60	144 (28.9%)
≥60	354 (71.1%)
Sex	
Female	255 (51.2%)
Male	243 (48.8%)
Tumor location	
Colon	362 (72.6%)
Rectum	136 (27.4%)
Local invasion	
T1-T2	107 (21.4%)
T3-T4	391 (78.6%)
Lymph node metastasis	
N0	303 (60.8%)
N1	108 (21.6%)
N2	87 (17.6%)
Distant metastasis	
M0	419 (84.1%)
M1	79 (15.9%)
TNM stage	
I	96 (19.2%)
II	197 (39.5%)
III	126 (25.3%)
IV	79 (16.0%)
Resection Margin Status	
R0	454 (91.1%)
R1	5 (1.0%)
R2	39 (7.9%)
Death	
No	413 (82.9%)
Yes	85 (17.0%)
